# A global review of publicly available datasets for ophthalmological imaging: barriers to access, usability, and generalisability

**DOI:** 10.1016/S2589-7500(20)30240-5

**Published:** 2020-10-01

**Authors:** Saad M Khan, Xiaoxuan Liu, Siddharth Nath, Edward Korot, Livia Faes, Siegfried K Wagner, Pearse A Keane, Neil J Sebire, Matthew J Burton, Alastair K Denniston

**Affiliations:** Academic Unit of Ophthalmology, Institute of Inflammation & Ageing, College of Medical and Dental Sciences, University of Birmingham, Birmingham, UK; Academic Unit of Ophthalmology, Institute of Inflammation & Ageing, College of Medical and Dental Sciences, University of Birmingham, Birmingham, UK; Ophthalmology Department, University Hospitals Birmingham NHS Foundation Trust, Birmingham, UK; Moorfields Eye Hospital NHS Foundation Trust, London, UK; Health Data Research UK, London, UK; Centre for Regulatory Science and Innovation, Birmingham Health Partners, Birmingham, UK; Department of Ophthalmology and Visual Sciences, McGill University, Montreal, QC, Canada; Moorfields Eye Hospital NHS Foundation Trust, London, UK; Stanford University Byers Eye Institute, Palo Alto, CA, USA; Moorfields Eye Hospital NHS Foundation Trust, London, UK; Eye Clinic, Cantonal Hospital of Lucerne, Lucerne, Switzerland; National Institute for Health Research Biomedical Research Centre for Ophthalmology, Moorfields Eye Hospital NHS Foundation Trust and UCL Institute of Ophthalmology, London, UK; National Institute for Health Research Biomedical Research Centre for Ophthalmology, Moorfields Eye Hospital NHS Foundation Trust and UCL Institute of Ophthalmology, London, UK; Health Data Research UK, London, UK; International Centre for Eye Health, Department of Clinical Research, London School of Hygiene & Tropical Medicine, London, UK; Academic Unit of Ophthalmology, Institute of Inflammation & Ageing, College of Medical and Dental Sciences, University of Birmingham, Birmingham, UK; Ophthalmology Department, University Hospitals Birmingham NHS Foundation Trust, Birmingham, UK; Health Data Research UK, London, UK; Centre for Regulatory Science and Innovation, Birmingham Health Partners, Birmingham, UK; National Institute for Health Research Biomedical Research Centre for Ophthalmology, Moorfields Eye Hospital NHS Foundation Trust and UCL Institute of Ophthalmology, London, UK

## Abstract

Health data that are publicly available are valuable resources for digital health research. Several public datasets containing ophthalmological imaging have been frequently used in machine learning research; however, the total number of datasets containing ophthalmological health information and their respective content is unclear. This Review aimed to identify all publicly available ophthalmological imaging datasets, detail their accessibility, describe which diseases and populations are represented, and report on the completeness of the associated metadata. With the use of MEDLINE, Google’s search engine, and Google Dataset Search, we identified 94 open access datasets containing 507 724 images and 125 videos from 122 364 patients. Most datasets originated from Asia, North America, and Europe. Disease populations were unevenly represented, with glaucoma, diabetic retinopathy, and age-related macular degeneration disproportionately overrepresented in comparison with other eye diseases. The reporting of basic demographic characteristics such as age, sex, and ethnicity was poor, even at the aggregate level. This Review provides greater visibility for ophthalmological datasets that are publicly available as powerful resources for research. Our paper also exposes an increasing divide in the representation of different population and disease groups in health data repositories. The improved reporting of metadata would enable researchers to access the most appropriate datasets for their needs and maximise the potential of such resources.

## Introduction

The availability of large, real world datasets has been essential in accelerating health data research, including the use of routinely collected data to drive new discoveries and innovations. Many of these innovations use advanced statistical and computational methods, such as machine learning (ML).^1,2^ These include the development of algorithms for the detection of breast cancer from mam mography, skin cancer from photographs, pneumonia from chest radiographs, and diabetic retinopathy from retinal fundus images, among many others.^3–7^ ML has also found many applications within ophthalmology, which include image segmentation, automated diagnosis, disease prediction, and prognostication. Ophthalmology is particularly suitable for ML because of the crucial role of imaging, where fundus photographs, optical coherence tomography (OCT), anterior segment photographs, and corneal topography can be applied to conditions such as diabetic retinopathy, age-related macular degeneration, glaucoma, papilloedema, and cataracts.^8–14^

High-quality health data research and the development of ML models requires meaningful data at a sufficient scale. Such data undoubtedly exist. Most health institutions hold clinical imaging data at a scale ranging from tens of thousands to tens of millions of scans. However, these data are often inaccessible to researchers, even where there is an intention to make them available for research, because of barriers of access and usability. Barriers of access can include: governance barriers (difficulties in understanding and working through governance frameworks regulating data usage); cost barriers (there can be considerable overhead costs to datasets and many datasets require payment for access); and time barriers (dataset requests and curation might incur a considerable time lag before they can be made available). Barriers of usability include: data format barriers (the data might not be in a computationally tractable form); data quality barriers (the data might be of insufficient or uncertain quality); and image labelling barriers (most imaging projects depend on the accurate labelling of those images, which might not be undertaken as part of routine care and are difficult to do retrospectively. To bypass these barriers, many research groups resort to using publicly available imaging datasets. This alternative route often leads to the same datasets within a clinical area being used by many research groups. Several wellknown public ophthalmological imaging datasets have been used multiple times by ML researchers, including MESSIDOR, DRIVE, EyePACS, and E-ophtha.^15–18^

Currently, there is no centralised directory of ophthalmological datasets and therefore little knowledge regard ing the amount of ophthalmological imaging data that are publicly available. It is also unclear what their accessibility is, and how complete the reporting is of associated metadata describing the image characteristics, the populations, and the diseases. This Review aims to identify all publicly available ophthalmological imaging datasets, to create a central directory of what is available for access currently. We report the source of each dataset, their accessibility, and a summary of the populations, diseases, and imaging types represented.

## Methods

### Search strategy and selection criteria

This Review forms part of *The Lancet Global Health* Commission on Global Eye Health, which is examining some of the central issues in global eye health.^19^ Any form of ophthalmological imaging was eligible for inclusion. Datasets containing non-ophthalmological images, text, or numerical-only data and images from non-human patients were excluded. No datasets were excluded on the basis of age, sex, or ethnicity of the patients from whom data were collected. Datasets of all languages and geographical origin were included.

The search consisted of two parts. First, we did a literature search of MEDLINE to identify studies de scribing ophthalmological imaging datasets that were publicly available and then attempted to access the datasets at source. The search combined terms describing various types of ophthalmological imaging, such as “eye”, “fundus”, and “retina”, with “optical coherence tomography”, “retinal images”, “biometry”, and “topography”, as well as terms such as “dataset” and “databases”. The MEDLINE search strategy is provided in the appendix (p 1). Second, we did a targeted search with similar search terms using Google’s Dataset Search and the Google search engine, to identify ophthalmological imaging datasets directly. Google’s Dataset Search is designed specifically for the discovery of online repositories and supports locating tabular, imaging, and text datasets. Additionally, indexing is provided to those interested in publicising their dataset with a metadata reference schema. For all the results returned from the search, it provides a description of the dataset content, direct links, and the file format. The Google searches also included terms relating to ocular diseases and ophthalmological imaging, and terms relating to datasets. For both the Google search and the Google Dataset searches, results returned from the first ten pages for each search were systematically collated and screened. The choice of ten pages was tested on the basis of several pilot searches at the beginning of this study to estimate the number of pages needed to capture the relevant results.

Our search was additionally supplemented by manually screening the references of relevant articles, the proceedings of relevant meetings, and consulting clinical experts in the field. The original search for all three sources, including all results from MEDLINE database inception, was done on Dec 3, 2019, and the MEDLINE search was updated on May 11, 2020. No restrictions were placed on language. Because of the absence of time-stamping for Google searches, we were unable to update the Google searches.

### Identification of ophthalmological imaging datasets

Search results from MEDLINE were screened by the primary reviewer (SMK) to identify the name and source of any relevant datasets. Where status of availability was unclear, we included these datasets and attempted to access their source, reporting any further barriers to access in our results. The Google and Google Dataset Search results were also screened by the primary reviewer to directly identify relevant datasets. Where there was ambiguity regarding whether a dataset fit the inclusion criteria, a second reviewer (XL) independently reviewed the dataset and if this could not be resolved, a third reviewer (SN) was consulted.

### Dataset access and description

Various classifications have been proposed for levels of data accessibility, including a tiered grading system where data accessibility is described as open, safe-guarded, or controlled.^20,21^ In this Review, we defined the accessibility of datasets as either: (1) open access, for which there were no requirements or minimum requirements for access (eg, submission of personal information, an email request, creation of an account); (2) open access with barriers, which were datasets fulfilling the theoretical criteria for open access, but being inaccessible because of unpredictable reasons (eg, no response to requests or broken hyperlinks); and (3) regulated access, which required the fulfilment of formal agreements, approvals, or payment. For the open access category where the dataset access required an email request, we allowed a 2-week period for email response; where the website was unresponsive or the download link was not functional, this was checked by a second and third reviewer, before the attempt to access was abandoned.

### Extraction of dataset characteristics

A prespecified data extraction form was developed and piloted on the first 20 datasets (by SMK and XL). Information characterising each dataset was recorded, including the direct links to the data source, accessibility, content (in terms of population, pathology, and imaging), and associated metadata (including clinical data, image labels, and segmentation). Where this information was reported at source (eg, on the dataset website) or a link to the paper describing the dataset was provided, we recorded the information as provided. Additionally, we presented the completeness of the reporting for key clinical metadata items across all open access datasets. Each item was marked as reported if the information was described in the dataset documentation, description, or referencing publication and was accepted as reported even if given at the aggregate level.

## Results

### Datasets identified from the literature search

**Figure 1:**
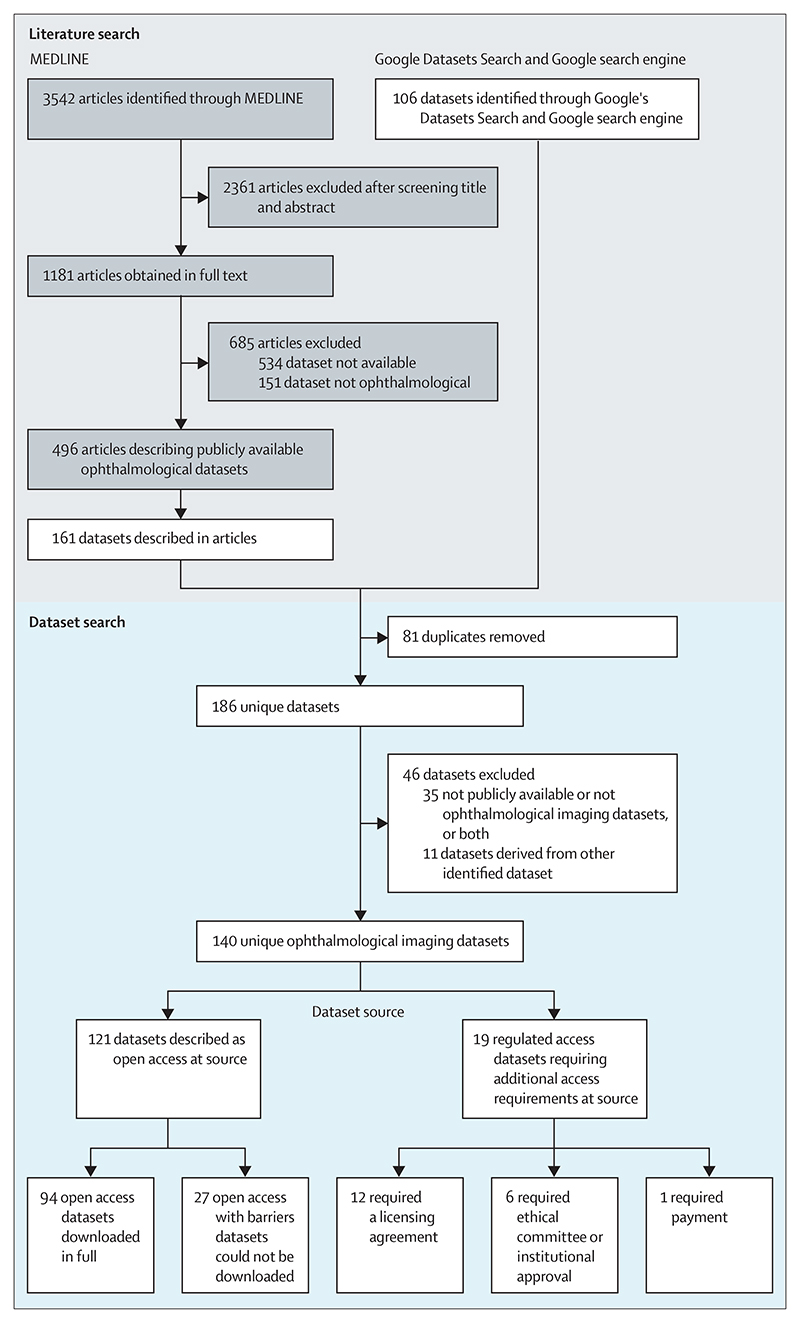
Dataset identification through MEDLINE articles, Google’s Datasets Search, and the Google search engine; and dataset selection and accessibility

The MEDLINE search identified 3542 articles, of which 2361 were deemed not relevant on the basis of screening their title and abstract. 1181 studies were obtained as full text articles, to be screened for the mention of ophthalmological imaging datasets that are publicly available. Of these, 534 records did not describe the datasets as publicly available and 151 reported non-ophthalmological datasets. Of the 496 articles that were still included, 161 potential datasets were identified. The same datasets were often referenced by multiple studies. The targeted search for datasets with the use of Google’s Dataset Search and Google search engine identified 106 datasets and after combining the results, 81 duplicate datasets were removed and 46 datasets were further excluded (35 not fitting the inclusion criteria and 11 were derived from other included datasets). 140 unique datasets were identified and included for further assessment at the data source. The dataset selection process is outlined in figure 1.

### Dataset access

Of the 140 unique datasets, only 94 were open access from which the raw data could be downloaded. 27 datasets were categorised as open access with barriers, from which data could not be downloaded. 19 datasets had regulated access (12 requiring licensing agreements, six requiring an ethical committee or institutional approval, and one requiring a payment of *£*2250 plus value-added tax).^22^ Only the 94 open access datasets could be thoroughly characterised from inspection of the raw data themselves. Details of the datasets in the open access with barriers and regulated access categories are included in the appendix (pp 2–3) and are derived from information found at the source or in the description of the dataset in its associated publication, or both, rather than inspection of the raw data itself.

### Characteristics of accessible datasets

**Figure 2:**
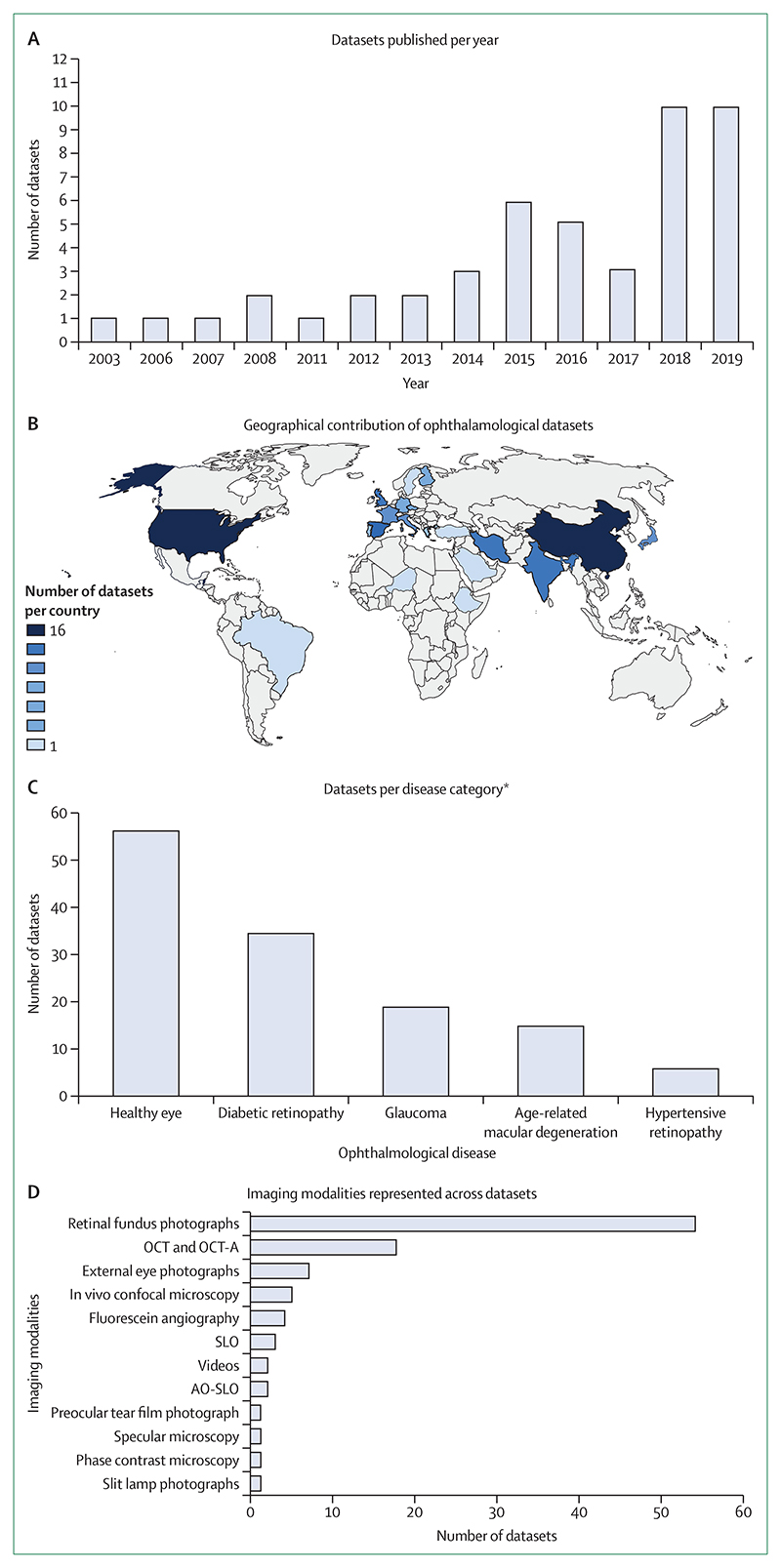
Information associated with the publication date (A), geographical distribution (B), represented diseases (C), and image types (D) of the study datasets AO-SLO=adaptive optics-scanning laser ophthalmoscopy. OCT=optical coherence tomography. OCT-A=optical coherence tomography-angiography. SLO=scanning laser ophthalmoscopy. *Only diseases represented in ≥5 datasets have been included. Where datasets included multiple diseases, they are counted multiple times.

Of the 94 open access datasets, we found 25 to be from within Asia (four from south Asia and 21 from southeast Asia or east Asia), nine from North Africa and the Middle East, 34 from Europe, 16 from North America, two from South America, and one from sub-Saharan Africa (figure 2). Of these, 9 datasets contained images originating from multiple countries. No data was reported to be originating from Oceania. The country of origin was unknown in 13 datasets. Dataset inception was reported by 47 datasets and ranged from 2003 to 2019. Open access dataset characteristics are summarised in the table.

From the 94 datasets, we were able to access 507 724 images and 125 videos from at least 122 364 patients (39 datasets did not record the number of patients). The number of patients across the datasets ranged from two to 85 550 (median=50; IQR=371), and the number of images ranged from eight to 109 312 (median=220; IQR=1017). The exact number of images could not be established for one dataset (CASIA Iris Ageing), so a conservative estimate was calculated from the dataset description (n=26 038). Of the total number of images, over half were contributed by three of the largest datasets: Kermany and colleagues (109 312 images),^5^ EyePACS (88 702 images),^23^ and MRL Eye (84 898 images).^24^ In contrast with these large datasets, 68 datasets had less than 1000 images, each ranging from eight to 850 images (median=111; IQR=245).

Where reported, the most common reason for image acquisition was for a research study or a clinical trial (54 of 94; 57%), and for routine clinical care or screening (23 of 94; 24%). Five of 94 (5%) datasets included images acquired from primary care (including screening programmes), 45 of 94 (48%) were from secondary care (hospital or eye clinics), 18 of 94 (19%) were collected in other settings (such as from a university, research settings, or eye banks), and one of 94 (1%) from a non-health-care setting. The setting was unreported in 25 of 94 (27%) datasets. Only 20 of 94 (21%) datasets gave information on whether patient consent was sought and 26 of 94 (28%) datasets stated details about obtaining ethical approval for obtaining or sharing the images.

Ophthalmological diseases represented in the datasets include diabetic eye disease (35 of 94; 37%), glaucoma (19 of 94; 20%), age-related macular degeneration (15 of 94; 16%), hypertensive retinopathy (six of 94; 6%), cataracts (four of 94; 4%), other eye diseases (the full list can be found in the table), and healthy eyes (58 of 94; 62%; figure 2). Moreover, 17 of 94 (18%) datasets did not specify the diseases represented in the dataset. It is possible that these datasets contained healthy eyes; however, no specific indication was given at the data source. 53 of 94 (56%) datasets contained more than one disease, including healthy eyes. Healthy eye images were intended for use in a range of biomedical applications, such as the analysis of normal anatomical structures (including endothelial cell density, the detection of photoreceptors, the assessment of nerves, and vessel morphology) and for technical uses (including denoising images, iris recognition, and eye tracking).

Imaging modalities included in the datasets included retinal fundus photographs (54 of 94; 57%), OCT or OCT angiography (18 of 94; 19%), external eye photographs (seven of 94; 7%), in vivo confocal microscopy (five of 94; 5%), scanning laser ophthalmoscopy and adaptive optics-scanning laser ophthalmoscopy (five of 94; 5%), fluorescein angiography (four of 94; 4%), slit lamp photographs (one of 94; 1%), phase contrast microscopy (one of 94; 1%), specular microscopy (one of 94; 1%), preocular tear film photograph (one of 94; 1%), and videos (two of 94; 2%; figure 2). Of these, five datasets contained images taken from more than one modality. Of the 18 imaging datasets based on OCT, half contained 2 dimensional imaging data and the other half contained 3 dimensional imaging data. Most datasets stored images in a portable network graphics, a tagged image file format, a bitmap image file, or a joint photographic experts group file format (82 of 94; 87%), ten of 94 (11%) datasets offered images as a Matlab file, one dataset offered a NumPy array file (Python), one dataset offered a portable pixmap format file (Netpbm) and 1 dataset offered a hierarchical data format file. Of these, nine of 94 (10%) datasets included images stored in multiple formats. 55 of 94 (59%) datasets included images annotated with labels (including diagnostic labels, eg, grade of diabetic retinopathy severity; or feature labels, eg, vessel labelled as an artery or vein) and 33 of 94 (35%) datasets included images annotated with manual segmentation, with 14 of 94 (15%) datasets providing both labels and segmentation. Annotations were provided by an array of experts including ophthalmology clinicians, general medical physicians, and researchers (including medical students, optometrists, operators, other undefined experts, or ground truth labellers). In addition, 15 datasets presented the images readily divided into either a training subset or a testing subset; however, they do not specify whether splits were made at the patient level.

### Completeness of metadata reporting

**Figure 3:**
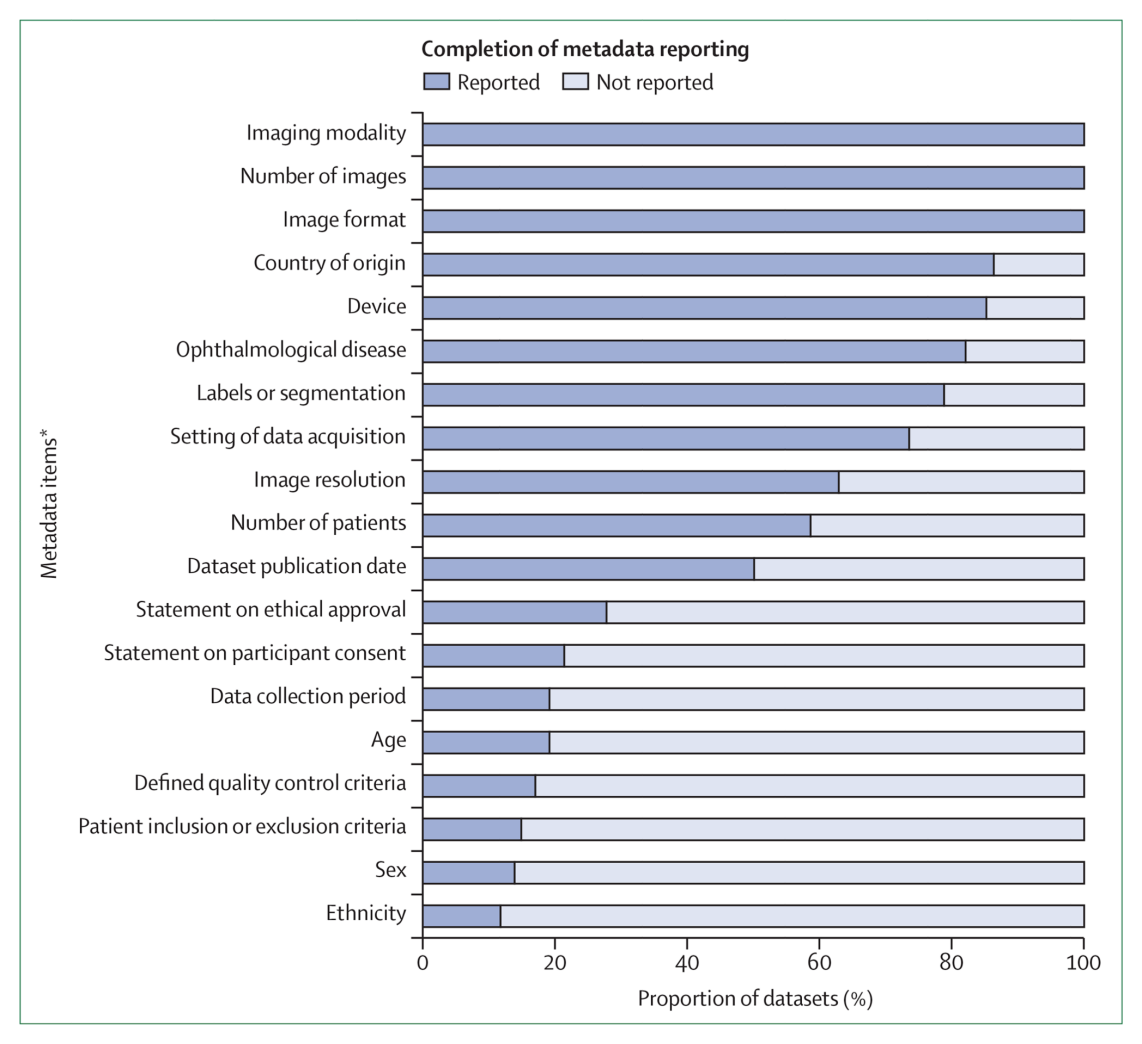
Percentage completion of reporting of metadata items across all 94 datasets *Reporting at the aggregate level was accepted.

The percentage of completion of the reporting for metadata items are shown in figure 3. Although technical details relating to the image files and their acquisition were well reported, any associated clinical information was not. The following information was consistently reported across all datasets: imaging modality (100%), number of images (100%), image format (100%), country of origin (86%), device name and manufacturer (85%), and ophthalmological disease (82%). Patient characteristics (including age, sex, and ethnicity) were particularly under-reported (these factors were reported in <20% of datasets), with 74% of the datasets not reporting any patient demographic data, even at the aggregate level. The inclusion and exclusion criteria were described for only 15% of the datasets and the data collection period was reported for only 19% of the datasets. The completeness of the metadata reported by each dataset is summarised in the appendix (pp 4–7).

## Discussion

### Summary of findings

To the best of our knowledge, this is the first study to curate a comprehensive list of ophthalmological imaging data that are publicly available. From a search of the medical literature and dataset search engines, our Review found 94 unique ophthalmological datasets that fit in the open access category, containing over 500 000 images. Besides healthy eyes, the most common diseases represented were diabetic retinopathy, glaucoma, and age-related macular degeneration. These diseases are most likely representative of the most commonly imaged diseases in routine clinical practice and research. In particular, screening programmes for diabetic retinopathy exist in several countries, leading to the accumulation of large national-level imaging data of the diabetic population.^25–27^

Across all datasets, fundus retinal photography was the most common imaging type (54 of 94 datasets), prob ably because of its widespread availability and common use across a wide range of ophthalmological diseases. The second most common imaging modality was OCT and OCT angiography (18 of 94 datasets, where 9 contained 3 dimensional OCT data). Preservation of the 3 dimensional volume data is advantageous as they give contextual information from neighbouring B-scans, allowing ML algorithms to learn key structural information that might enhance its performance.

Several variables that are clinically essential were under-reported across all datasets. Demographic data including age, sex, and ethnicity were not reported in most of the datasets (74%), even at the aggregate level. Furthermore, inclusion and exclusion criteria were only defined for 15% of the datasets. This missing information is a concern as it is unclear whether there is appropriate representation of population groups within the data, and the ability of researchers to assess the applicability to research findings from these data will be severely restricted.

For datasets with image labels (such as diagnostic or feature labels), the labelling processes were also poorly defined. Many assumptions are made during the labelling of ground truths, and therefore assurance regarding the label accuracy are paramount since they carry implications for any ML model trained with the use of these labels. Details about the labellers’ amount of expertise, the consensus process used for multiple labellers, and how discrepancies were resolved are therefore all relevant.^28^ In the few datasets that reported this information, labellers ranged from medical students to specialist ophthalmologists, but in most cases the skills of the labellers were unknown. Although the detailed labelling of public datasets might be ambitious, a checklist of minimum reporting metadata items could drastically improve the usefulness of the data and could also potentially enable merging across multiple datasets.

### Strengths and weaknesses

This is the first study to systematically identify ophthalmological imaging datasets that are publicly available. An important aspect of the work is the unrestrictive nature of the search strategy applied to a medical bibliographic dataset and online search engines, including those specifically targeting datasets. This method recognises the possibility that not all relevant datasets would be identified with the use of academic publications alone. Furthermore, we sought to verify the claims of all datasets, so that we could adjudge the extent to which such datasets were truly open access and what the user experience might be. We took reasonable measures to obtain actual copies of the data, so their contents could be examined and verified. This process gave us the ability to identify accessibility barriers where data described as being open access were difficult to access regardless. This process also enabled us to identify the extent to which key metadata were available.

We recognise several limitations to our study. Firstly, only the initial ten pages of results returned by a Google Dataset search and Google’s search engine results were screened. It is not clear how the ranking of datasets in Google Datasets Search is established, but according to the documentation, metadata quality, the number of citations, and a combination of other factors are taken into account.^29^ The nature of the Google search engines is such that it is not possible to update the searches, whereas the MEDLINE search was originally run in December, 2019 and could be updated in May, 2020. Given the probable delays between a dataset being publicly available, and a study being completed and becoming visible on MEDLINE, it would be reasonable to assume that our Review describes the situation as of December, 2019. Secondly, we recognise that this field is moving fast and that this Review is only a snapshot in time. Unlike publications, datasets can be edited, updated, and removed without documentation. As with the timestamping issue, these alterations prohibit the ability to monitor changes in data availability over time. Thirdly, there are other sources of datasets available, such as Kaggle, but these were not explored in this Review (although the Google Dataset Search tool explicitly indexes Kaggle). Future iterations of this study will explore other search engines beyond Google Datasets Search. Lastly, our aim was specifically targeted towards identifying open access datasets. As such, some datasets with regulated access might not have been identified if they were not described as being open access. If there were requirements for accessing the data, we took reasonable measures to obtain actual copies. We emailed the authors or owners of the data (when requested) but did not go as far as completing licensing agreements or pursuing any ethical committee approvals. Although verification of access for those datasets would be welcome, doing so was outside the scope of this Review and would have imposed an additional burden on those institutions with little value other than simple verification. However, we do recognise that the datasets with regulated access might be of higher quality, and that this more restricted access might reflect governance processes associated with a stronger attention to quality and metadata reporting. Regardless, as noted earlier, ease and speed of access is an important driver for researchers, and there is a risk that the benefits of a higher quality dataset might be overlooked in favour of a lower quality dataset that has immediate, unregulated access.

### Implications

Publicly available imaging datasets are increasingly being used by a range of researchers from epidemiologists to computer scientists. These datasets can be a powerful enabler to research but, as with any data source, the provenance and limitations of that dataset must be considered. In this section, we highlight three broad implications for the providers and users of such datasets: accessibility; transparency and reporting; and ensuring an adequate representation of the population.

The first implication is accessibility. It is encouraging that our Review identified 94 datasets that were potentially open access, but discoverability appears to be an issue. Although a few datasets are well known, many are not, which might lead to lost research opportunities and might result in bias because of an overuse of a few potentially non-representative datasets. There is value in having an online catalogue of such datasets, which would improve their visibility and provide some key metadata that would enable researchers to identify the most suitable dataset for their research question. Our study provides an initial point of access that will improve their discoverability. Further considerations in this regard, arising from our Review, include a greater clarity around the terms of access from some dataset providers. Although it was beyond the scope of this study, such datasets should also be accompanied by sufficient information regarding their provenance so that researchers can be assured that there is an appropriate ethical and governance framework underpinning the provision of these data.

The second important implication is the transparency and reporting of a dataset. The value of a dataset is associated with far more than just its size, and our Review has highlighted many factors that would be key considerations for a user. There are, of course, advantages to scale, for example in the development of deep learning models or when seeking to detect a modest signal in a heterogeneous population, but the usability of the dataset will also be associated with the quality, depth, and representativeness of the data. Small datasets such as DRIVE (consisting of only 40 images) are examples of a situation in which high-quality labelling and annotation outweighs quantity. DRIVE has become a popular resource for researchers for the purposes of retinal vessel segmentation, probably because of the richness and quality of the segmentation annotations.^30,31^ Given the need for researchers to show the generalisability of research findings and their clinical applicability, it is essential that these digital repositories are adequately representative of the diverse population of humans and their diseases. Important characteristics should be re ported to assist the user in decisions around applicability. There is sparse reporting of data characteristics and little guidance to inform the curators of such datasets. Although these might be unimportant considerations from a technical perspective, they are crucial to consider for any clinical applications. Without key information about the population and disease, it is impossible to make assumptions on how generalisable the data are for a real world setting. Previous work outside of the field of health data, such as Datasheets for Datasets (a concept derived from the electronics industry), have previously highlighted many of the issues raised in this Review, which are prevalent across disciplines.^32^ Gebru and colleagues^32^ have proposed the reporting of considerations that can improve the transparency and accountability of datasets.

However, there are recognised challenges associated with providing richly labelled data. The curation of metadata items is demanding, costly, and requires careful consideration to ensure accuracy and completeness. The excessive inclusion of detailed metadata could also increase the chance of the reidentification of data items and pose additional privacy concerns. Therefore curation, storage, and access all require thoughtful ethical oversight. However, these risks should be balanced with the potential harm implicated by widespread use of biased and clinically unusable data. Additionally, the risk of reidentification can be mitigated with adherence to widely adopted guidelines for the sharing of raw clinical trial data.^33^ The investment of time, skill, and money would generate substantial value in the data and its associated labels, therefore such a dataset is unlikely to be freely available.

The last key implication is around ensuring adequate representation of the population by such datasets. A major concern is the possibility of the underrepresentation of specific groups within public and other datasets, posing unknown biases towards some populations or disease groups. An ML algorithm developed exclusively on one population group might translate poorly beyond that population.^34^ If an ML algorithm runs poorly on unseen data that are inadequately described, it is difficult to establish whether the poor performance is attributable to spectrum bias.^35–37^ Knowledge of the populations represented is therefore important for the development of ML algorithms and even more so for their evaluation. This is a key consideration from a global perspective, as countries wishing to develop applications where there is no infrastructure to curate imaging datasets might also be most likely to access publicly available resources as a first option.

Underrepresentation of diseases is also a concern. Datasets are likely to reflect diseases of particular relevance to their country of origin; data from routine clinical care will reflect the prevalence of the disease of the attending population; and cohort data will reflect the inclusion criteria of the study, but also the health priorities of that particular country. The type of diseases within each dataset and their prevalence within the dataset will affect the generalisability of that dataset to other settings globally. It is important to note that, of the priority eye diseases highlighted by WHO, only diabetic retinopathy, glaucoma, and age-related macular degeneration were strongly represented in the public imaging datasets. For 2015, these three conditions together were estimated to account for 15% of global blindness and 5% of moderate and severe vision impairment, in contrast with the other priority diseases such as cataracts (four datasets), trachoma (one dataset), and refractive errors (three datasets), which contribute to 53% of blindness and 79% of moderate and severe vision impairment.^38^ This mismatch might be attributed to many factors, including the relative importance of imaging in the management of the disease, the presence of well developed screening programmes for the most represented diseases (such as diabetic retinopathy) and funding available for specific research areas. Diabetic retinopathy, glaucoma, and age-related macular degeneration are more frequently imaged as part of standard care, as opposed to cataracts, trachoma, and refractive errors. If potential imaging-based solutions could improve the care of patients with cataracts, trachoma, and refractive errors by a non-specialist workforce with the use of task sharing, then perhaps a targeted global effort is required to prioritise the curation and development of imaging in these disease areas.

The publicly available datasets identified in this Review are unevenly distributed globally. There are no known publicly available datasets for ophthalmological images in 172 countries (equating to nearly 3·5 billion people, or 45% of the global population). The availability of needed data is even lower if specific use cases are considered. For understanding the healthy eye, there are 58 datasets from 20 countries, representing 54% of the population; for age-related macular degeneration, there are 15 datasets from 6 countries, representing an estimated 44% of the population; for diabetic eye disease, there are 35 datasets from 14 countries, representing 50% of the population. Inferences from data cannot be assumed to generalise across populations and might be unusable on unseen populations. We would argue that this is a form of data poverty that should be taken seriously as it might cause widening of health inequalities, as major parts of the world are unable to benefit from innovations arising in a small pool of data-rich countries.

## Conclusion

Publicly available datasets are potentially valuable assets for research and innovation in health care. Barriers to their use include poor visibility, issues of accessibility, or limited usability because of incomplete metadata, includ ing an absence of key parameters necessary for evaluating the provenance, quality of data, and the diversity of the population sampled. There is a danger that research ers use a small, skewed pool of data because there are only a few datasets that have high visibility, along with easy access and usability. In real world evidence studies this might lead to substantial bias. In the deployment of artifical intelligence systems it might lead to a poor generalisability, with a risk of underperformance or even failure when transferred between settings and groups of people. We propose that this is a new form of data poverty, where the scarce availability of representative datasets (public and other) will restrict the extent to which some individuals or even whole populations can benefit from digital health solutions and artifical intelligence systems. Here lies an opportunity to not only improve the visibility, accessibility, and usability of existing publicly available datasets, but also for health systems and researchers to invest in new publicly available datasets that can support research, innovation, and validation in areas that currently have few data.

## Supplementary Material

Supplementary appendix

## Figures and Tables

**Table 1:** Characteristics of the open access datasets

	Access type	Data access details	Link to dataset	Country of origin	Number of patients	Number of images	Eye diseases	File type	Device (manufacturer)	File format
ACRIMA	OA	Downloadable zip file	https://figshare.com/s/c2d31f850af14c5b5232	Spain	NR	705	Glaucoma and healthy eyes	Fundus photograph	TRC retina camera (Topcon, Japan)	JPEG
Dataset for AO-SLO cone photoreceptor automatic segmentation and analysis; 2013 Chiu	OA	Downloadable zip file	http://people.duke.edu/~sf59/software.html	NR	21	840	Healthy eye and one patient with deuteranopia	AO-SLO	Confocal adaptive optics scanning laser ophthalmoscope (unspecified)	MAT
Asia Pacific Tele-Ophthalmology Society	OA	Create Kaggle account to download zip file	https://www.kaggle.com/c/aptos2019-blindness-detection/data	India	NR	5590	Diabetic retinopathy and healthy eyes	Fundus photograph	Fundus camera (unspecified)	PNG
Arteriovenous Nicking	OA	Downloadable zip file	https://people.eng.unimelb.edu.au/thivun/projects/AV_nicking_quantification/	NR	NR	90	NR	Fundus photograph	NR	PNG
BioMediTech	OA	Downloadable zip file	https://figshare.com/s/d6fb591f1beb4f8efa6f	NR	NR	195	NR	Phase contrast microscopy	Eclipse TE200S phase contrast microscope (Nikon, Japan)	TIFF
CASIA Iris Ageing*	OA	Create account to download zip file	http://biometrics.idealtest.org/findTotalDbByMode.do?mode=Iris	China	50	26 038	NR	External iris photograph	H100 (IrisGuard, UK), AD100 (IrisGuard), and Irispass-h (OKI, Japan)	BMP
CASIA Iris Image Version 4	OA	Create account to download zip file	http://biometrics.idealtest.org/findTotalDbByMode.do?mode=Iris	China	1800	54 601	NR	External iris photograph	Close-up iris camera (CASIA, China), IRISPASS-h (OKI, Japan), long-range iris camera (CASIA), and IKEMB-100 (IrisKing, China)	JPEG
CASIA Iris Mobile	OA	Create account to download zip file	http://biometrics.idealtest.org/findTotalDbByMode.do?mode=Iris	China	630	11 000	NR	External iris photograph	Near-infrared iris imaging module v1 and v2 (CASIA) and a domestic mobile telephone with near-infrared iris-scanning technology (CASIA)	JPEG
Retina	OA	Create Kaggle account to download zip file	https://www.kaggle.com/jr2ngb/cataractdataset	NR	NR	601	Glaucoma, cataracts, retinal diseases, healthy eyes	Fundus photograph	NR	PNG
Cataract-101	OA	Downloadable zip file	https://doi.org/10.5281/zenodo.1220951	Austria	101	101	Cataracts	Video of cataract surgery	Camera (unspecified)	MPEG-4 part 14
Child Heart Health Study in England	OA	Downloadable zip file	https://blogs.kingston.ac.uk/retinal/chasedb1/	UK	14	28	Healthy eyes	Fundus photograph	NM-200D handheld fundus camera (Nidek, Japan)	JPEG
2014 Srinivasan	OA	Downloadable zip file	http://people.duke.edu/~sf59/software.html	USA	45	3231	Diabetic eye disease, age-related macular degeneration, healthy eyes	OCT	Heidelberg SPECTRALIS SD-OCT imaging system (Heidelberg Engineering, Germany)	TIFF
Contact Lens Anterior Segment*-*Optical Coherence Tomography Understanding Dataset	AoR	Give email address to receive a link to download zip file	http://www.varpa.es/research/ophtalmology.html#cloud	Spain	16	112	NR	OCT	OCT Cirrus 500 scanner (Carl Zeiss Meditec, Germany) with an anterior segment module for users of scleral contact lens	JPEG
Cone Detection	OA	Downloadable zip file	https://github.com/DavidCunefare/CNN-Cone-Detection	USA	18	264	Healthy eyes	AO-SLO	Split detector adaptive optics scanning laser ophthalmoscope (unspecified)	TIFF
Rotterdam Ophthalmic Data Repository Cornea	OA	Downloadable zip file	http://www.rodrep.com/data-sets.html	Netherlands	23	52	Fuchs’ endothelial corneal dystrophy	In vivo confocal microscopy	ConfoScan 4 confocal microscope (Nidek Technologies, Italy)	PNG
Corneal Endothelial Cell	OA	Downloadable zip file	https://github.com/daboe01/SREP-18-33533B	NR	385	385	Diseased corneas and healthy eyes	Specular microscopy corneal endothelial cells photograph	SP-3000 specular microscope (Topcon)	TIFF
Corneal Heidelberg OCT	OA	Download data from Google Drive	https://sites.google.com/site/hosseinrabbanikhorasgani/datasets-1/corneal-oct	Iran	15	579	Healthy eyes	OCT	Heidelberg SPECTRALIS OCT HRA system (Heidelberg Engineering)	MAT
Corneal 3D Reconstruction	OA	Fill in a form and get emailed a link to download zip file	http://bioimlab.dei.unipd.it/Data%20Sets.htm	Italy	3	356	NR	In vivo confocal microscopy	ConfoScan 4 confocal microscope (Nidek Technologies)	JPEG
Corneal Nerve	OA	Fill in a form and get emailed a link to download zip file	http://bioimlab.dei.unipd.it/Data%20Sets.htm	Italy	90	90	Healthy eyes	In vivo confocal microscopy	ConfoScan 4 confocal microscope (Nidek Technologies)	JPEG
Corneal Nerve Tortuosity	OA	Fill in a form and get emailed a link to download zip file	http://bioimlab.dei.unipd.it/Data%20Sets.htm	Japan	30	30	Diabetes, pseudoexfoliation syndrome, keratoconus, healthy eyes	In vivo confocal microscopy	Heidelberg retina tomograph II with rostock corneal module (Heidelberg Engineering)	TIFF
Retinal Fundus and OCT	OA	Downloadable zip file	https://sites.google.com/site/hosseinrabbanikhorasgani/datasets-1/vessel-reg-oct-fundus	Iran	22	44	Various retinal diseases	OCT and fundus photograph	3D OCT-1000 (Topcon)	MAT (OCT) and JPEG (Fundus)
2013 Fang	OA	Downloadable zip file	http://people.duke.edu/~sf59/software.html	USA	13	195	Healthy eyes	OCT	SD-OCT imaging system (Bioptigen, USA)	TIFF
2012 Fang	OA	Downloadable zip file	http://people.duke.edu/~sf59/software.html	USA	17	51	Age-related macular degeneration and healthy eyes	OCT	SD-OCT imaging system (Bioptigen)	TIFF
Digital Extraction from Retinal Images of Veins and Arteries	OA	Downloadable zip file	https://medicine.uiowa.edu/eye/abramoff/	Netherlands	50	50	Diabetic eye disease and healthy eyes	Fundus photograph	Topcon NW100 (Topcon) and Canon CR5-45NM (Canon, Japan), non-mydriatic colour fundus cameras	TIFF
Standard Diabetic Retinopathy Database Calibration Level 0	OA	Downloadable zip file	http://www.it.lut.fi/project/imageret/diaretdb0/	Finland	NR	130	Diabetic eye disease and healthy eyes	Fundus photograph	Digital fundus camera (unspecified)	PNG
Standard Diabetic Retinopathy Database Calibration Level 1	OA	Downloadable zip file	http://www.it.lut.fi/project/imageret/diaretdbl/index.html	Finland	NR	89	Diabetic eye disease and healthy eyes	Fundus photograph	Digital fundus camera (unspecified)	PNG
Diabetic Retinopathy, Hypertension, Age-related Macular Degeneration and Glaucoma Images	OA	Downloadable zip file	https://personalpages.manchester.ac.uk/staff/niall.p.mcloughlin/	UK	38	39	Diabetic eye disease, hypertensive retinopathy, glaucoma, age-related macular degeneration	Fundus photograph	TRC-NW6s (Topcon), TRC-NW8 (Topcon), or CR-DGi fundus camera (Canon)	JPEG and PNG
DR1	OA	Downloadable zip file	https://figshare.com/articles/Advancing_Bag_of_Visual_Words_Representations_for_Lesion_Classification_in_Retinal_Images/953671/2	Brazil	NR	1077	Diabetic eye disease and healthy eyes	Fundus photograph	TRC-50X mydriatic camera (Topcon)	TIFF
DR2	OA	Downloadable zip file	https://figshare.com/articles/Advancing_Bag_of_Visual_Words_Representations_for_Lesion_Classification_in_Retinal_Images/953671/3	Brazil	NR	520	Diabetic eye disease and healthy eyes	Fundus photograph	TRC-NW8 retinograph (Topcon) with a D90 camera (Nikon, Japan)	TIFF
Diabetic Retinopathy Image Database	OA	Download each image separately	http://isbb.ktu.edu.tr/multimedia/drimdb/	Turkey	NR	216	NR	Fundus photograph	CF-60UVi fundus camera (Canon)	JPEG
Digital Retinal Images for Optic Nerve Segmentation	OA	Downloadable zip file	http://www.ia.uned.es/~ejcarmona/DRIONS-DB.html	Spain	55	110	Hypertensive retinopathy, glaucoma	Fundus photograph	Colour analogical fundus camera (unspecified)	JPEG
Drishti-GS1	OA	Register by filling out a form to download zip file	http://cvit.iiit.ac.in/projects/mip/drishti-gs/mip-dataset2/Home.php	India	NR	101	Glaucoma and healthy eyes	Fundus photograph	NR	PNG
Digital Retinal Images for Vessel Extraction	OA	Create account to download zip file	https://www.isi.uu.nl/Research/Databases/DRIVE/	Netherlands	400	40	Diabetic eye disease and healthy eyes	Fundus photograph	CR5 non-mydriatic 3CCD camera (Canon)	JPEG
Duke OCT	OA	Downloadable files from Dropbox	http://people.duke.edu/~sf59/RPEDC_Ophth_2013_dataset.htm	USA	384	38 400	Age-related macular degeneration and healthy eyes	OCT	SD-OCT imaging system (Bioptigen)	MAT
Glaucoma Fundus	OA	Downloadable zip file	https://dataverse.harvard.edu/dataset.xhtml?persistentId=doi:10’791/DVN/1YRRAC	South Korea	1542	1542	Glaucoma and healthy eyes	Fundus photograph	AFC-330 non-mydriatic auto fundus camera (Nidek)	PNG
E-ophtha	OA	Fill in a form and get emailed a code to download zip file	http://www.adcis.net/en/third-party/e-ophtha/	France	NR	463	Diabetic eye disease and healthy eyes	Fundus photograph	NR	JPEG
Eye Picture Archive Communication System	OA	Create Kaggle account to download zip file	http://www.eyepacs.com/data-analysis	USA	NR	88 702	Diabetic eye disease	Fundus photograph	Centervue DRS (Centervue, Italy), Optovue iCam (Optovue, USA), Canon CR1/DGi/CR2 (Canon), and Topcon NW (Topcon)	JPEG
2015 Rabbani	OA	Download zip file from Dropbox	http://people.duke.edu/~sf59/software.html	USA	24	24 images and 24 videos	Diabetic eye disease	Fundus fluorescein angiogram photograph and videos	Heidelberg SPECTRALIS OCT HRA system (Heidelberg Engineering)	TIFF
Fundus Image Registration Dataset	OA	Downloadable zip file	https://www.ics.forth.gr/cvrl/fire/	Greece	39	268	NR	Fundus photograph	AFC-210 fundus camera (Nidek)	JPEG
Fundus Fluorescein Angiogram and Colour Fundus	OA	Downloadable zip file	https://sites.google.com/site/hosseinrabbanikhorasgani/datasets-5	Iran	60	120	Diabetic eye disease and healthy eyes	Fundus fluorescein angiogram photograph and fundus photograph	NR	JPEG
Fundus Fluorescein Angiogram	OA	Downloadable zip file	https://sites.google.com/site/hosseinrabbanikhorasgani/datasets-3	Iran	70	70	Diabetic eye disease and healthy eyes	Fundus fluorescein angiogram photograph	NR	JPEG
Fundus Images with Exudates	OA	Downloadable zip file	https://sites.google.com/site/hosseinrabbanikhorasgani/datasets-1/fundus-images-with-exudates	Iran	NR	35	Diabetic eye disease	Fundus photograph	NR	JPEG
Hamilton Eye Institute Macular Edema	OA	Downloadable zip file	https://github.com/lgiancaUTH/HEI-MED	USA	910	169	Diabetic eye disease and healthy eyes	Fundus photograph	Visucam PRO fundus camera (ZEISS, Germany)	JPEG
High-Resolution Fundus Quality Assessment	OA	Downloadable zip file	https://www5.cs.fau.de/research/data/fundus-images/	Germany and Czech Republic	18	36	NR	Fundus photograph	CR-1 fundus camera (Canon)	JPEG
High-Resolution Fundus Segmentation	OA	Downloadable zip file	https://www5.cs.fau.de/research/data/fundus-images/	Germany and Czech Republic	45	45	Diabetic eye disease, glaucoma, healthy eyes	Fundus photograph	CF-60UVi camera (Canon)	JPEG
iChallenge age related macular degeneration	OA	Create BAIDU account to download zip files	http://ai.baidu.com/broad/introduction	China	NR	1200	Age-related macular degeneration and healthy eyes	Fundus photograph	NR	JPEG
iChallenge Pathological Myopia	OA	Create BAIDU account to download zip files	https://ichallenges.grand-challenge.org/iChallenge-AMD/	China	NR	1200	Myopia and healthy eyes	Fundus photograph	Visucam 500 fundus camera (ZEISS)	JPEG
Indian Diabetic Retinopathy Image Dataset	OA	Create BAIDU account to download zip files	https://idrid.grand-challenge.org/Rules/	India	NR	516	Diabetic eye disease and healthy eyes	Fundus photograph	VX-10 alpha digital fundus camera (Kowa, USA)	JPEG
Iowa Normative Set for Processing Images of the Retina— Arteriovenous ratio	OA	Fill in a form and get emailed a link to download zip file	https://medicine.uiowa.edu/eye/inspire-datasets	USA	NR	40	Glaucoma	Fundus photograph	Fundus camera (Carl Zeiss Meditec)	JPEG
Iowa Normative Set for Processing Images of the Retina—Stereo	OA	Fill in a form and get emailed a link to download zip file	https://medicine.uiowa.edu/eye/inspire-datasets	USA	15	30	Glaucoma	Stereo fundus photograph	3Dx digital stereo retinal camera (Nidek)	TIFF
IOSTAR Retinal Vessel	OA	Register to download zip file	http://www.retinacheck.org/download-iostar-retinal-vessel-segmentation-dataset	Netherlands and China	NR	30	NR	SLO	EasyScan camera (i-Optics, Netherlands)	JPEG
Jichi DR	OA	Downloadable zip file	https://journals.plos.org/plosone/article?id=10’1371/journal.pone.0179790#sec006	Japan	2740	9939	Diabetic eye disease and healthy eyes	Fundus photograph	AFC-230 fundus camera (Nidek)	JPEG
Joint Shantou International Eye Centre	OA	Downloadable zip file	https://www.kaggle.com/linchundan/fundusimage1000	China	NR	1000	Long list of diseases 1 (see end of table)	Fundus photograph	NR	JPEG
Kermany/Guangzhou	OA	Downloadable zip file	https://data.mendeley.com/datasets/rscbjbr9sj/3	USA and China	5319	109 312	Diabetic eye disease, drusen, choroidal neovascularisation, healthy eyes	OCT	Heidelberg SPECTRALIS SD-OCT imaging system (Heidelberg Engineering)	JPEG
Large-scale Attention-based Glaucoma	AoR	Email authors for password to access files on Dropbox	https://github.com/smilell/AG-CNN	China	NR	4854	Glaucoma and healthy eyes	Fundus photograph	NR	JPEG
Rotterdam Ophthalmic Data Repository DR	OA	Downloadable zip file	http://www.rodrep.com/data-sets.html	Netherlands	70	1120	Diabetic eye disease	Fundus photograph	TRC-NW65 non-mydriatic digital fundus camera (Topcon)	PNG
Messidor-2f	OA	Fill in a form and get emailed a code to download zip file	http://www.adcis.net/en/third-party/messidor2/	France	874	1748	Diabetic eye disease	Fundus photograph	TRC-NW6 non-mydriatic fundus camera (Topcon)	JPEG and PNG
Miles Iris	OA	Downloadable zip file	https://drive.google.com/drive/folders/OB5OBp4zckpLnYkpBcWlubCOtcTA	NR	NR	832	NR	External iris photograph	NR	JPEG
MRL Eye	OA	Downloadable zip file	http://mrl.cs.vsb.cz/eyedataset	NR	37	84 898	Healthy eyes	External eye photograph	Intel RealSense RS 300 (Intel, USA), IDS Imaging sensor (IDS Imaging Development Systems, Germany), and Aptina sensor (Aptina Imaging, USA)	PNG
Noor Hospital	OA	Need password to decrypt images in the downloadable zip file	https://drive.google.com/file/d/1iSiFfD5LpLASrFUZu13uMFSRcFEjvbSq/view	Iran	148	4142	Diabetic eye disease, age-related macular degeneration, healthy eyes	OCT	Heidelberg SPECTRALIS SD-OCT imaging system (Heidelberg Engineering)	TIFF
2015 Chiu	OA	Downloadable zip file	http://people.duke.edu/~sf59/software.html	USA	10	10	Diabetic eye disease	OCT	Heidelberg SPECTRALIS SD-OCT imaging system (Heidelberg Engineering)	MAT
Healthy OCT and Fundus	OA	Need password to access images in the downloadable zip file	https://sites.google.com/site/hosseinrabbanikhorasgani/datasets-1/oct-fundus-right-left	NR	50	100	Healthy eyes	OCT and fundus photograph	3D OCT Topcon device (Topcon)	MAT and JPEG
OCT Glaucoma Detection	OA	Downloadable zip file	https://zenodo.org/record/1481223#.XrO6Q2gzbIU	NR	624	1100	Glaucoma and healthy eyes	OCT	Cirrus SD-OCT scanner (ZEISS)	NumPy Array File
OCTAGON	AoR	Give email address to receive a link, username, and password to download zip file	http://www.varpa.es/research/ophtalmology.html	Spain	213	213	Diabetic eye disease and healthy eyes	OCT angiography	DRI OCT Triton (Topcon)	JPEG and TIFF
Optical Coherence Tomography Retinal Image Analysis 3D	OA	Downloadable zip file	https://journals.plos.org/plosone/article?id=10’1371/journal.pone.0133908#secOO2	NR	10	10	Healthy eyes	OCT	Heidelberg SPECTRALIS SD-OCT imaging system (Heidelberg Engineering)	MAT
Ocular Disease Intelligent Recognition	OA	Join competition and wait for request approval to download dataset	https://odir2019.grand-challenge.org/Download/	China	5000	8000	Diabetic eye disease, hypertensive retinopathy, glaucoma, age-related macular degeneration, cataracts, myopia, other diseases, healthy eyes	Fundus photograph	Fundus camera (Canon), Fundus camera (ZEISS), and Fundus camera (Kowa)	JPEG
Optic Nerve Head Segmentation Dataset	OA	Downloadable zip file	http://www.aldiri.info/Image%20Datasets/ONHSD.aspx	UK	50	99	Diabetic eye disease	Fundus photograph	CR6 45MNf fundus camera (Canon)	BMP
Ophthalmic Slit Lamp	OA	Downloadable zip file	https://plos.figshare.com/articles/Predicting_the_progression_of_ophthalmic_disease_based_on_slit-lamp_images_using_a_deep_temporal_sequence_network/6883823	China	NR	60	Cataracts	Slit lamp photograph	Slit Lamp (unspecified)	JPEG
Canada OCT Retinal Images	OA	Downloadable zip file	https://dataverse.scholarsportal.info/dataverse/OCTID	India	NR	470	Diabetic eye disease, age-related macular degeneration, macular hole, central serous retinopathy, healthy eyes	OCT	Cirrus HD-OCT machine (Carl Zeiss Meditec)	JPEG
Online Retinal Fundus Image Dataset for Glaucoma Analysis and Research—650	OA	Downloadable zip file	https://drive.google.com/drive/folders/1VPCvVsPgrfPNIl932xgU3XC_WFLUsXJR	Singapore	3280	650	Glaucoma and healthy eyes	Fundus photograph	NR	JPEG
Project MACulopathy Unveiled by Laminar Analysis	OA	Download every image separately	https://link.springer.com/article/104007%2Fs11517-018-1915-z#Sec2	USA	NR	239	Age-related macular degeneration and healthy eyes	OCT and fundus photograph	Digital camera (Nikon) and Heidelberg SPECTRALIS OCT HRA system (Heidelberg Engineering)	JPEG
Corneal Nerve Plexus	OA	Downloadable zip file	https://figshare.com/collections/SBP_Mosaic_Dataset/3950197	Sweden	82	164	Diabetic eye disease and healthy eyes	In vivo confocal microscopy	Laser-scanning in vivo confocal microscopy (unspecified)	TIFF
RetinaCheck-Microaneurysm	OA	Register for link to download zip file	http://www.retinacheck.org/download-iostar-retinal-vessel-segmentation-dataset	Netherlands and China	NR	250	NR	Fundus photograph	DRS non-mydriatic fundus camera (Centervue, Italy)	JPEG
RetinaCheck-Scanning Laser Ophthalmoscopy vessel patch	OA	Register for link to download zip file	http://www.retinacheck.org/download-iostar-retinal-vessel-segmentation-dataset	Netherlands and China	NR	40	NR	SLO	EasyScan camera (i-Optics)	TIFF
RetinaCheck-Scanning Laser Ophthalmoscopy-Microaneurysm	OA	Register for link to download zip file	http://www.retinacheck.org/download-iostar-retinal-vessel-segmentation-dataset	Netherlands and China	NR	58	NR	SLO	EasyScan camera (i-Optics)	TIFF
Retinal Fundus Glaucoma Challenge	OA	Create BAIDU account to download zip files	https://ai.baidu.com/broad/download?dataset=gon	China	NR	1200	Glaucoma and healthy eyes	Fundus photograph	Visucam 500 fundus camera (ZEISS) and Canon CR-2 camera (Canon)	JPEG
Retinal Vessel Tortuosity	OA	Fill in a form and get emailed a link to download zip file	http://bioimlab.dei.unipd.it/Data%20Sets.htm	Italy	34	60	Hypertensive retinopathy and healthy eyes	Fundus photograph	TRC 50 fundus camera (Topcon)	JPEG
Retinal Vessel Image set for Estimation of Widths	OA	Downloadable zip file	http://www.aldiri.info/Image%20Datasets/Review.aspx	UK	NR	16	Diabetic eye disease	Fundus photograph	Canon 60 UV film camera (Canon), Fundus camera (ZEISS), and JVC 3CCD (JVC, Japan)	JPEG and BMP
Retinal fundus Images for Glaucoma Analysis	OA	Downloadable zip file	https://deepblue.lib.umich.edu/data/concern/data_sets/3b591905z?locale=en	Saudi Arabia and France	NR	750	Glaucoma and healthy eyes	Fundus photograph	Mydriatic and non-mydriatic retinal camera (unspecified)	TIFF and JPEG
RIM-ONE Version 2	OA	Downloadable zip file	http://medimrg.webs.ull.es/	Spain	NR	455	Glaucoma and healthy eyes	Fundus photograph	AFC-210 fundus camera (Nidek) with a body of Canon EOS 5D Mark II (Canon)	JPEG
RIM-ONE Version 3	OA	Downloadable zip file	http://medimrg.webs.ull.es/	Spain	NR	159	Glaucoma and healthy eyes	Stereo fundus photograph	AFC-210 fundus camera (Nidek) with a body of Canon EOS 5D Mark II (Canon)	JPEG
Retina Online Challenge	OA	Fill in a form and get emailed a link to download zip file	http://webeye.ophth.uiowa.edu/ROC/	Netherlands	NR	100	Diabetic eye disease	Fundus photograph	TRC-NW100 (Topcon), TRC-NW200 (Topcon), or CR5–45NM (Canon)	JPEG
Retinal Optical Coherence Tomography Classification Challenge	OA	Downloadable zip file	https://rocc.grand-challenge.org/Participation/	Iran	NR	165	Diabetic eye disease and healthy eyes	OCT	SD-OCT device (Topcon)	MAT
Investigative Ophthalmology & Visual Science; 2011 Chiu	OA	Downloadable zip file	http://people.duke.edu/~sf59/software.html	USA	20	220	Age-related macular degeneration	OCT	SD-OCT imaging system (Bioptigen)	MAT
Structured Analysis of the Retina	OA	Downloadable zip file	http://cecas.clemson.edu/~ahoover/stare/	USA	NR	397	Long list of diseases 2 (see end of table)	Fundus photograph	TRV-50 fundus camera (Topcon)	Portable Pixmap Format
Trachoma	OA	Downloadable zip file	https://doi.org/10.6084/m9.figshare.7551053.v1	Niger and Ethiopia	85 550	1656	Trachoma and healthy eyes	Conjunctival photograph	Single-lens reflex camera (unspecified)	JPEG
Tsukazaki Hospital	OA	Enter name, email address, and affiliation to receive link and password for dataset download from Dropbox	https://tsukazaki-ai.github.io/optos_dataset/	Japan	5389	13 047	Diabetic eye disease, glaucoma, age-related macular degeneration, retinal vein occlusion, macular hole, retinal detachment, retinitis pigmentosa, artery occlusion, diabetes, healthy eyes	Fundus photograph	200Tx ultrawide-field device (Optos, UK)	JPEG
University of Palackeho and Olomouc Iris	OA	Downloadable zip file	http://www.cbsr.ia.ac.cn:8080/iapr_database.jsp	Czech Republic	64	384	NR	External iris photograph	TRC50IA optical device (Topcon) connected with DXC-950P 3CCD camera (Sony, Japan)	PNG
Vampire	OA	Downloadable zip file	https://vampire.computing.dundee.ac.uk/vesselseg.html	NR	2	8	Age-related macular degeneration and healthy eyes	Ultrawide fundus fluorescein angiogram photograph	P200C retinal imaging (Optos, UK)	PNG and BMP
VARPA images for the computation of the arterio/venular ratio	AoR	Give email address to receive a link, username, and password to download zip file	http://www.varpa.es/research/ophtalmology.html	Spain	NR	58	NR	Fundus photograph	TRC-NW100 non-mydriatic camera (Topcon)	JPEG
VARPA optical dataset	AoR	Email to get username and password to download zip file	http://www.varpa.es/research/optics.html#databases	NR	NR	128	Healthy eyes	Preocular tear film photograph	Tearscope Plus (Keeler, UK)	BMP, JPEG, and PNG
WIDE	OA	Downloadable zip file	http://people.duke.edu/~sf59/software.html	USA	30	30	Age-related macular degeneration and healthy eyes	Fundus photograph	200Tx ultrawide-field device (Optos)	MAT
William Hoyt	OA	Download each image separately	https://novel.utah.edu/Hoyt/	NR	NR	850	Papilloedema, pseudo-papilledema, disc swelling from local and systemic causes, congenital anomalies of the optic disc, optic atrophy, retinocerebral diseases	Fundus photograph	NR	JPEG
Yangxi	OA	Downloadable zip file	https://zenodo.org/record/3393265#.XazZaOgzbIV	China	5825	18 394	Age-related macular degeneration and healthy eyes	Fundus photograph	Non-mydriatic digital fundus camera (Crystalvue, Taiwan)	Hierarchical Data Format

AoR=Available on request. AO-SLO=Adaptive optics-scanning laser ophthalmoscopy. BMP=Bitmap image file. CASIA=Institute of Automation, Chinese Academy of Sciences. DR=Diabetic Retinopathy. JPEG=Joint photographic experts group. MAT=Matlab. NR=Not reported. OA=Open access. OCT=Optical Coherence Tomography. PNG=Portable network graphics. SLO=Scanning laser ophthalmoscopy. TIFF=Tagged image file format. *Number of images were estimated on the basis of a conservative number from the dataset description. †Messidor-2 is an updated version of the original Messidor dataset. Long list of diseases 1: diabetic eye disease, hypertensive retinopathy, glaucoma, tessellated fundus, large optic cup, optic atrophy, disc swelling and elevation, dragged disc, congenital disc abnormality, retinitis pigmentosa, biette crystalline dystrophy, peripheral retinal degeneration, myelinated nerve fibre, vitreous particles, fundus neoplasm, branch retinal vein occlusion, central retinal vein occlusion, massive hard exudates, yellow white spot flecks, cottonwool spots, vessel tortuosity, chorioretinal atrophy-coloboma, preretinal haemorrhage, fibrosis, laser spots, silicon oil in eye, blur fundus without proliferative diabetic retinopathy, blur fundus with suspected proliferative diabetic retinopathy, retinal artery occlusion, rhegmatogenous retinal detachment, central serous chorioretinopathy, Vogt-Koyanagi-Harada disease, maculopathy, epiretinal membrane, macular hole, pathological myopia, healthy eyes. Long list of diseases 2: diabetic eye disease, hypertensive retinopathy, age-related macular degeneration, choroidal neovascularisation, Hollenhorst Emboli, branch retinal artery occlusion, cilio-retinal artery occlusion, branch retinal vein occlusion, central retinal vein occlusion, hemi-central retinal vein occlusion, arteriosclerotic retinopathy, coat’s, macroaneurysm, histoplasmosis, nevus, epiretinal membrane, drusen, retinitis, toxoplasmosis, choroidal melanoma, myelinated nerve fibres, optic nerve atrophy, stellate maculopathy, chorioretinal scar, frosted branch vasculopathy, asteroid hyalosis, vasculitis, patterned retinal pigmented epitheliopathy, choroidal hemangioma, unknown diagnosis, healthy eyes.
