# Supplementary material for: A global review of publicly available datasets for ophthalmological imaging: barriers to access, usability, and generalisability
Source: Lancet Digit Health. Author manuscript; Available in PMC 2025 Oct 22. (PMC7618278; doi:10.1016/S2589-7500(20)30240-5)
Supplement: Supplementary appendix [file EMS209644-supplement-Supplementary_appendix.pdf]

THE LANCET
Digital Health

Supplementary appendix

This appendix formed part of the original submission and has been peer reviewed. We post it as supplied by the authors.

Supplement to: Khan SM, Liu X, Nath S, et al. A global review of publicly available datasets for ophthalmological imaging: barriers to access, usability, and generalisability. *Lancet Digit Health* 2020; published online Oct 1. https://doi.org/10.1016/S2589-7500(20)30240-5.

SUPPLEMENTARY INFORMATION

Supplementary Table 1: Search strategy for the MEDLINE database

1. exp Dataset/
2. exp Databases, Factual/
3. exp "Neural Networks (Computer)"/ or exp Artificial Intelligence/
4. 1 or 2 or 3
5. optic*.mp.
6. ophthalm*.mp.
7. exp Eye/
8. 5 or 6 or 7
9. image.mp.
10. imaging.mp.
11. 9 or 10
12. 8 and 11
13. exp Biometry/
14. 8 and 13
15. exp Diagnostic Techniques, Ophthalmological/
16. retina.mp. or exp Retina/
17. exp Retinal Diseases/
18. retinal images.mp. or exp Retinal Vessels/
19. exp Fundus Oculi/ or fundus.mp.
20. exp Optic Disk/ or fundus images.mp.
21. exp Tomography, Optical Coherence/ or oct.mp.
22. Slit lamp imaging.mp.
23. Slit lamp photography.mp.
24. exp Visual Fields/
25. Perimetry.mp.
26. exp Corneal Topography/
27. exp Fluorescein Angiography/
28. exp Electroretinography/
29. B scan.mp.
30. Ocular ultrasound.mp.
31. Retinal photography.mp.
32. exp Indocyanine Green/
33. Anterior segment photography.mp.
34. Fundus autofluorescence.mp.
35. Optic nerve head analysis.mp.
36. exp Microscopy, Confocal/
37. Scanning laser ophthalmoscopy.mp.
38. External Eye Photography.mp.
39. Specular microscopy.mp.
40. Optical coherence tomography angiography.mp.
41. Adaptive optics.mp.
42. Iris angiography'.mp.
43. Hess Chart.mp.
44. Microperimetry.mp.
45. Nuclear lacrimal scintigraphy.mp.
46. Dacryocystography.mp.
47. 12 or 14 or 15 or 16 or 17 or 18 or 19 or 20 or 21 or 22 or 23 or 24 or 25 or 26 or 27 or 28 or 29 or 30 or 31 or 32 or 33 or 34 or 35 or 36 or 37 or 38 or 39 or 40 or 41 or 42 or 43 or 44 or 45 or 46
48. 4 and 47

Supplementary Table 2: Characteristics of the "open access with barriers" and "regulated access" (*in grey*) datasets

Dataset Name	Barrier to Access	Link to Dataset	Country of Origin*	Number of Images*	Image Type*	Diseases included*
Capillary Nonperfusion Analysis	No response to email request	https://cvit.iiit.ac.in/research/projects/cvit-projects/retinal-image-analysis?showall=&start=1	India			
AMD Analysis	No response to email request	https://cvit.iiit.ac.in/research/projects/cvit-projects/retinal-image-analysis?showall=&start=1	India			
Collection of Fundus Multispectral Images	No response to email request	https://www.cs.bham.ac.uk/research/projects/fundus-multispectral/	UK		Fundus photograph	Healthy eyes
BIOMISA Retinal Image Database	No response to email request	http://biomisa.org/index.php/downloads/	Pakistan	2,561	Fundus photograph and OCT	Glaucoma, AMD; Macular Oedema and Healthy eyes
BIOMISA AMD	No response to email request	http://biomisa.org/index.php/downloads/	Pakistan	6,900	Fundus photograph and OCT	AMD and Healthy eyes
BIOMISA Hypertensive Retinopathy	No response to email request	http://biomisa.org/index.php/downloads/	Pakistan	100	Fundus photograph	Diabetic Eye Disease; Hypertensive Retinopathy; Macular Oedema; Papilledema
BIOMISA Glaucoma Fundus	No response to email request	http://biomisa.org/index.php/downloads/	Pakistan	462	Fundus photograph	Glaucoma and Healthy eyes
BIOMISA Glaucoma Fundus and OCT	No response to email request	http://biomisa.org/index.php/downloads/	Pakistan	132	Fundus photograph and OCT	Glaucoma and Healthy eyes
BIOMISA Retina Identification	No response to email request	http://biomisa.org/index.php/downloads/	Pakistan	100	Fundus photograph	Healthy eyes
OPTIMA	Dataset not downloadable as challenge is closed	https://optima.meduniwien.ac.at/research/challenges/		30	OCT	
LabelMe	Website under maintenance	http://www.labelme.org/#				
VARIA	No response to email request	http://www.varpa.es/research/biometrics.html#databases	Spain	233		
Automatic Retinal Image Analysis	Website not responding	https://www.eyecharity.com/aria_online/	UK	161	Fundus photograph	AMD and Healthy eyes
Retinal OCT Fluid Challenge	Unable to download dataset as access not given and unresponsive link to data confidentiality agreement	https://retouch.grand-challenge.org/Home/		112	OCT	AMD; Retinal Vein Occlusion
Optic neuropathies and Pseudopapilledema	No response to email request	https://www.ncbi.nlm.nih.gov/pmc/articles/PMC6688269/	South Korea	1,369	Fundus photograph	Diabetic Eye Disease; Optic Neuropathies; Pseudopapilledema and Healthy eyes
Open EDS: Open Eye	No response to email request	https://research.fb.com/programs/openeds-challenge		356,649	External eye photograph	
Paediatric Cataract	No response to email request	https://translational-medicine.biomedcentral.com/articles/10.1186/s12967-018-1758-2#Sec2	China		Slit lamp photograph	Cataracts
Bath Iris	Download link not working	http://www.cbsr.ia.ac.cn:8080/iapr_database.jsp	UK	1,000	External iris photograph	
Visual Field	No response to email request	https://www.ncbi.nlm.nih.gov/pmc/articles/PMC6172715/	China	4,012	Visual fields	Glaucoma; Cataracts and Healthy eyes
Diabetic Retinopathy Image Dataset	No response to email request	https://ipg.fer.hr/ipg/resources/image_database	Croatia	50	Fundus photograph	Diabetic Eye Disease and Healthy eyes
KNHANES	Enter email address but does not allow to download data	https://knhanes.cdc.go.kr/knhanes/eng/index.do				
Cataracts Challenge	No dataset download link found	https://cataracts.grand-challenge.org/	France	50 videos	Video	Cataracts
Paediatric Cataract Programme	No response to email request	https://biomedical-engineering-online.biomedcentral.com/articles/10.1186/s12938-017-0420-1#Sec2	China	2,705	Slit lamp photograph	Cataracts
Detection of diabetic retinopathy in primary care offices	No response to email request	https://www.nature.com/articles/s41746-018-0040-6#Sec1	USA		Fundus photograph and OCT	Diabetic Eye Disease and Healthy eyes
Hong Kong OCT	No response to email request	https://www.sciencedirect.com/science/article/pii/S2589750019300858#cesec100	China	4,877	OCT	Glaucoma and Healthy eyes
LMD-DRS	No response to Google form application	https://home.deec.uc.pt/~lcruz/lmd/	Portugal	622	Fundus photograph	Diabetic Eye Disease; Laser Marks and Healthy eyes
LMD-BAPT	No response to Google form application	https://home.deec.uc.pt/~lcruz/lmd/	Portugal	49	Fundus photograph	Laser Marks
ND Iris 0405	License agreement required	https://cvrl.nd.edu/projects/data/#nd-iris-0405-data-set	USA	64,980	External eye photograph	
ND CrossSensor Iris 2013	License agreement required	https://cvrl.nd.edu/projects/data/#nd-iris-0405-data-set	USA	146,550	External eye photograph	
ND Cosmetic Contact Lenses 2013	License agreement required	https://cvrl.nd.edu/projects/data/#nd-iris-0405-data-set	USA	5,100	External eye photograph	Subjects without contact lenses, with soft contact lenses, and with cosmetic contact lenses
ND LivDet Iris 2017	License agreement required	https://cvrl.nd.edu/projects/data/#nd-iris-0405-data-set	USA	4,800	External eye photograph	Subjects with and without contact lenses

Multiple Biometric Grand Challenge Version 2	License agreement required	https://cvrl.nd.edu/projects/data/#nd-iris-0405-data-set	USA		External eye photograph	
ND Contact Lenses Dataset 2015	License agreement required	https://cvrl.nd.edu/projects/data/#nd-iris-0405-data-set	USA	7,300	External eye photograph	
ND Iris Template Aging 2008-2010	License agreement required	https://cvrl.nd.edu/projects/data/#nd-iris-0405-data-set	USA	11,776	External eye photograph	
ND Iris Contact Lenses 2010	License agreement required	https://cvrl.nd.edu/projects/data/#nd-iris-0405-data-set	USA	21,700	External eye photograph	
ND Gender from Iris Dataset	License agreement required	https://cvrl.nd.edu/projects/data/#nd-iris-0405-data-set	USA	3,000	External eye photograph	
UBIRIS Version 2	License agreement required	http://iris.di.ubi.pt/	Portugal	11,102	External eye photograph	
Taiwan Private Hospital	Need permission from institution	https://www.ncbi.nlm.nih.gov/pmc/articles/PMC6101083/	Taiwan		Fundus photograph	Diabetic Eye Disease and Healthy eyes
Mobbio Iris	License agreement required	https://paginas.fe.up.pt/~mobbio2013/				
University of Auckland Retinopathy	License agreement required	https://figshare.com/articles/UoA-DR_Database_Info/5985208	India	200	Fundus photograph	Diabetic Eye Disease and Healthy eyes
Age-Related Eye Disease Study	Data access request needed	https://www.ncbi.nlm.nih.gov/projects/gap/cgi-bin/study.cgi?study_id=phs000001.v3.p1&phv=53743&phd=1&pha=2856&pht=371&phvf=&phdf=&phaf=&phtf=&dssp=1&consent=&temp=1	USA	206,500	Fundus photograph	AMD; Cataracts and Healthy eyes
UK Biobank	Need to register, apply, sign material transfer agreement, and submit payment	https://blogs.kingston.ac.uk/retinal/uk-biobank/	UK	135,867	Fundus photograph	
KORA	Individual project agreement needed with KORA	https://epi.helmholtz-muenchen.de/	Germany		Fundus photograph	AMD and Healthy eyes
Moorfields	Ethical approval required	https://www.nature.com/articles/s41591-018-0107-6	UK	16,990	OCT	Diabetic Eye Disease; AMD; Choroidal Neovascularisation; Multiple Retinal Pathologies and Healthy eyes
Rajavithi Hospital	Ethical approval required	https://www.nature.com/articles/s41467-019-13922-8#Sec9	Thailand	7,072	Fundus photograph	Diabetic Eye Disease and Healthy eyes
Yang OCT	Institutional approval required	https://journals.plos.org/plosone/article?id=10.1371/journal.pone.0215076	South Korea	10,100	OCT	AMD and Healthy eyes

*****blank fields due to data not being reported or not accessible. **Abbreviations - AMD:** Age-related macular degeneration**, BIOMISA:** BioMedical Image and Signal Analysis, **KNHANES:** Korea National Health and Nutrition Examination Survey, **KORA:** Kooperative Gesundheitsforschung in der Region Augsburg, **LMD-DRS:** Laser Marks Dataset - Diabetic Retinopathy Screening, **LMD-BAPT:** Laser Marks Dataset - Before and After Photocoagulation Treatment, **ND:** Notre Dame, **OCT:** Optical Coherence Tomography**, UK:** United Kingdom, **USA:** United States of America, **VARIA:** VARPA Retinal images for authentication

Supplementary Table 3: Reporting of metadata for each "open access" study dataset

Dataset	Setting of Data Acquisition	Country of Origin	Dataset Publication Date	Statement on Participant Consent	Statement on Ethical Approval	Number of Patients	Age	Gender	Ethnicity	Ophthalmic Disease	Patient Inclusion/ Exclusion Criteria	Data Collection Period	Number of Images	Imaging Modality	Device	Image Format	Image Resolution	Defined Quality Control Criteria	Labels/Segmentation
ACRIMA	Y	Y	Y	Y	Y	N	N	N	N	Y	N	N	Y	Y	Y	Y	N	Y	Y
Dataset for AO-SLO cone photoreceptor automatic segmentation and analysis (Chiu 2013)	Y	N	N	Y	Y	Y	Y	Y	N	Y	N	N	Y	Y	Y	Y	Y	N	Y
APTOS	Y	Y	Y	N	N	N	N	N	N	Y	N	N	Y	Y	Y	Y	N	N	Y
AV Nicking	N	N	Y	N	N	N	N	N	N	N	N	N	Y	Y	N	Y	N	N	Y
BioMediTech	N	N	Y	N	Y	N	N	N	N	N	N	N	Y	Y	Y	Y	Y	N	Y
CASIA Iris Ageing	Y	Y	N	N	N	Y	N	N	N	N	N	Y	Y	Y	Y	Y	N	N	N
CASIA Iris Image Version 4	Y	Y	N	N	N	Y	N	N	N	N	N	N	Y	Y	Y	Y	Y	N	N
CASIA Iris Mobile	Y	Y	N	N	N	Y	N	N	Y	N	N	N	Y	Y	Y	Y	Y	N	N
Retina	N	N	Y	N	N	N	N	N	N	Y	N	N	Y	Y	N	Y	N	N	Y
Cataract-101	Y	Y	Y	N	N	Y	N	N	N	Y	Y	N	Y	Y	N	Y	Y	N	Y
CHASE	Y	Y	N	Y	Y	Y	Y	N	Y	Y	N	N	Y	Y	Y	Y	Y	N	Y
2014 Srinivasan	Y	Y	Y	N	Y	Y	N	N	N	Y	N	N	Y	Y	Y	Y	N	Y	Y
CLOUD	N	Y	N	Y	Y	Y	N	N	N	N	N	N	Y	Y	Y	Y	N	N	N
Cone Detection	N	Y	Y	N	N	Y	N	N	N	Y	N	N	Y	Y	Y	Y	Y	N	Y
ROD-REP Cornea	Y	Y	N	Y	Y	Y	N	N	N	Y	Y	N	Y	Y	Y	Y	Y	N	Y
Corneal Endothelial Cell	N	N	Y	N	Y	Y	N	N	N	Y	N	N	Y	Y	Y	Y	N	N	Y
Corneal Heidelberg OCT	Y	Y	Y	N	N	Y	N	N	N	Y	N	N	Y	Y	Y	Y	Y	N	N
Corneal 3D Reconstruction	N	Y	N	N	N	Y	N	N	N	N	N	N	Y	Y	Y	Y	Y	N	N
Corneal Nerve	N	Y	N	N	N	Y	N	N	N	Y	N	N	Y	Y	Y	Y	Y	N	Y
Corneal Nerve Tortuosity	Y	Y	N	N	N	Y	N	N	N	Y	N	N	Y	Y	Y	Y	Y	N	Y
Retinal Fundus and OCT	N	Y	N	N	N	Y	N	N	N	Y	N	N	Y	Y	Y	Y	N	N	N
2013 Fang	Y	Y	Y	Y	Y	Y	N	N	N	Y	Y	N	Y	Y	Y	Y	N	N	N
2012 Fang	Y	Y	Y	Y	N	Y	N	N	N	Y	Y	Y	Y	Y	Y	Y	N	N	N
DERIVA	Y	Y	N	Y	N	Y	N	N	N	Y	Y	Y	Y	Y	Y	Y	Y	Y	N
DiaRetDB0	Y	Y	Y	N	N	N	N	N	N	Y	N	N	Y	Y	Y	Y	Y	Y	Y
DiaRetDB1	Y	Y	Y	N	N	N	N	N	N	Y	N	N	Y	Y	Y	Y	Y	N	Y
DR HAGIS	Y	Y	Y	Y	N	Y	N	N	N	Y	N	N	Y	Y	Y	Y	Y	N	Y
DR1	Y	Y	Y	N	N	N	N	N	N	Y	N	N	Y	Y	Y	Y	Y	N	Y
DR2	Y	Y	Y	N	N	N	N	N	N	Y	N	N	Y	Y	Y	Y	Y	N	Y

DRIMDB	Y	Y	Y	N	N	N	N	N	N	N	N	N	Y	Y	Y	Y	Y	Y	Y
DRIONS	Y	Y	Y	N	N	Y	Y	Y	Y	Y	N	N	Y	Y	Y	Y	Y	Y	Y
Drishti-GS1	Y	Y	Y	Y	N	N	Y	Y	Y	Y	N	N	Y	Y	N	Y	Y	Y	Y
DRIVE	Y	Y	N	N	N	Y	Y	N	N	Y	N	N	Y	Y	Y	Y	Y	N	Y
Duke OCT	Y	Y	N	Y	Y	Y	Y	N	N	Y	Y	Y	Y	Y	Y	Y	N	Y	Y
Glaucoma Fundus	Y	Y	Y	N	Y	Y	N	N	N	Y	Y	N	Y	Y	Y	Y	Y	N	Y
E-ophtha	Y	Y	N	N	N	N	N	N	N	Y	N	Y	Y	Y	N	Y	N	N	Y
EyePACS	Y	Y	N	N	N	N	N	N	N	Y	N	Y	Y	Y	Y	Y	N	N	Y
2015 Rabbani	Y	Y	Y	N	Y	Y	N	N	N	Y	Y	N	Y	Y	Y	Y	Y	Y	Y
FIRE	Y	Y	N	Y	N	Y	Y	N	N	N	N	Y	Y	Y	Y	Y	Y	N	Y
Fundus Fluorescein Angiogram and Colour Fundus	Y	Y	N	N	N	Y	N	N	N	Y	N	N	Y	Y	N	Y	Y	N	Y
Fundus Fluorescein Angiogram	Y	Y	N	N	N	Y	N	N	N	Y	N	N	Y	Y	N	Y	Y	N	Y
Fundus Images with Exudates	N	Y	N	N	N	N	N	N	N	Y	N	N	Y	Y	N	Y	Y	N	N
HEI Macular Edema	Y	Y	N	N	N	Y	Y	Y	Y	Y	N	Y	Y	Y	Y	Y	Y	Y	Y
HRF Quality Assessment	Y	Y	N	N	N	Y	N	N	N	N	N	N	Y	Y	Y	Y	N	N	N
HRF Segmentation	Y	Y	N	N	N	Y	N	N	N	Y	N	N	Y	Y	Y	Y	Y	N	Y
iChallenge AMD	N	Y	N	N	N	N	N	N	N	Y	N	N	Y	Y	N	Y	N	N	Y
iChallenge PM	N	Y	N	N	N	N	N	N	N	Y	N	N	Y	Y	Y	Y	N	N	Y
IDRiD	Y	Y	Y	N	N	N	N	N	Y	Y	N	N	Y	Y	Y	Y	Y	Y	Y
INSPIRE-AV ratio	Y	Y	N	N	N	N	N	N	N	Y	N	N	Y	Y	Y	Y	Y	N	N
INSPIRE-Stereo	Y	Y	N	N	N	Y	N	N	N	Y	N	N	Y	Y	Y	Y	Y	N	N
IOSTAR Retinal Vessel	N	Y	Y	N	N	N	N	N	N	N	N	N	Y	Y	Y	Y	Y	N	Y
Jichi DR	Y	Y	Y	Y	Y	Y	N	N	N	Y	N	Y	Y	Y	Y	Y	Y	N	Y
JSIEC	Y	Y	Y	N	N	N	N	N	N	Y	N	N	Y	Y	N	Y	N	N	N
Kermany/ Guangzhou	Y	Y	Y	N	Y	Y	N	N	N	Y	Y	Y	Y	Y	Y	Y	N	Y	Y
LAG	Y	Y	Y	Y	N	N	N	N	N	Y	N	N	Y	Y	N	Y	Y	N	Y
ROD-REP DR	Y	Y	N	N	Y	Y	Y	Y	N	Y	Y	Y	Y	Y	Y	Y	Y	N	N
Messidor-2*	Y	Y	N	N	N	Y	N	N	N	Y	N	Y	Y	Y	Y	Y	Y	N	Y
Miles Iris	N	N	Y	N	N	N	N	N	N	N	N	N	Y	Y	N	Y	N	N	N
MRL Eye	Y	N	N	N	N	Y	N	Y	N	Y	N	N	Y	Y	Y	Y	Y	N	Y
Nor Hospital	Y	Y	Y	N	N	Y	N	N	N	Y	N	N	Y	Y	Y	Y	Y	N	Y
2015 Chiu	Y	Y	Y	N	N	Y	N	N	N	Y	N	N	Y	Y	Y	Y	N	N	Y
Healthy OCT and Fundus	N	N	N	N	N	Y	N	N	N	Y	N	N	Y	Y	Y	Y	N	N	N
OCT Glaucoma Detection	N	N	Y	Y	Y	Y	Y	Y	Y	Y	N	N	Y	Y	Y	Y	N	N	Y
OCTAGON	N	Y	N	N	Y	Y	Y	N	N	Y	N	N	Y	Y	Y	Y	Y	N	Y

OCTRIMA 3D	N	N	N	Y	Y	Y	N	N	N	Y	Y	N	Y	Y	Y	Y	N	N	Y
ODIR	Y	Y	Y	N	N	Y	Y	Y	N	Y	N	N	Y	Y	Y	Y	N	Y	Y
ONHSD	Y	Y	N	N	N	Y	Y	Y	Y	Y	N	N	Y	Y	Y	Y	Y	N	Y
Ophthalmic Slit Lamp	Y	Y	Y	Y	Y	N	N	N	N	Y	N	N	Y	Y	Y	Y	N	N	N
Canada OCT Retinal Images	Y	Y	Y	N	N	N	N	N	N	Y	N	N	Y	Y	Y	Y	Y	N	Y
ORIGA-650	Y	Y	N	N	N	Y	N	N	Y	Y	N	Y	Y	Y	N	Y	N	N	Y
Project Macula	Y	Y	N	N	Y	N	Y	Y	Y	Y	N	N	Y	Y	Y	Y	N	N	Y
Corneal Nerve Plexus	Y	Y	Y	Y	Y	Y	Y	N	N	Y	Y	Y	Y	Y	Y	Y	N	N	Y
RC-RGB-MA	N	Y	N	N	N	N	N	N	N	N	N	N	Y	Y	Y	Y	Y	N	Y
RC-SLO vessel patch	N	Y	N	N	N	N	N	N	N	N	N	N	Y	Y	Y	Y	Y	N	Y
RC-SLO-MA	N	Y	N	N	N	N	N	N	N	N	N	N	Y	Y	Y	Y	Y	N	Y
REFUGE	Y	Y	Y	N	N	N	N	Y	Y	Y	N	N	Y	Y	Y	Y	Y	Y	Y
Retinal Vessel Tortuosity	Y	Y	N	N	N	Y	N	N	N	Y	N	N	Y	Y	Y	Y	Y	N	Y
REVIEW	Y	Y	N	N	N	N	N	N	N	Y	N	N	Y	Y	Y	Y	Y	N	Y
RIGA	Y	Y	Y	N	N	N	N	N	N	Y	N	N	Y	Y	Y	Y	Y	N	Y
RIM-ONE Version 2	Y	Y	Y	N	Y	N	N	N	N	Y	N	N	Y	Y	Y	Y	N	N	Y
RIM-ONE Version 3	Y	Y	Y	N	Y	N	N	N	N	Y	N	N	Y	Y	Y	Y	N	N	Y
ROC	Y	Y	Y	N	N	N	N	N	N	Y	Y	Y	Y	Y	Y	Y	Y	N	Y
ROCC	Y	Y	N	N	N	N	N	N	N	Y	N	N	Y	Y	Y	Y	N	N	Y
2011 IOVS Chiu	Y	Y	N	Y	N	Y	N	N	N	Y	Y	Y	Y	Y	Y	Y	N	Y	Y
STARE	Y	Y	N	N	N	N	N	N	N	Y	N	N	Y	Y	Y	Y	Y	N	Y
Trachoma	Y	Y	Y	Y	Y	Y	N	Y	N	Y	N	N	Y	Y	Y	Y	Y	N	Y
Tsukazaki Hospital	Y	Y	Y	N	Y	Y	Y	Y	N	Y	N	Y	Y	Y	Y	Y	N	N	Y
UPOL Iris	Y	Y	Y	N	N	Y	N	N	N	N	N	N	Y	Y	Y	Y	Y	N	N
Vampire	N	N	Y	N	N	Y	N	N	N	Y	N	N	Y	Y	Y	Y	Y	N	Y
VICAVR	Y	Y	N	N	N	N	N	N	N	N	N	N	Y	Y	Y	Y	Y	N	Y
VOPTICAL	N	N	N	N	N	N	Y	N	N	Y	N	N	Y	Y	Y	Y	Y	N	Y
WIDE	Y	Y	Y	N	N	Y	N	N	N	Y	N	Y	Y	Y	Y	Y	Y	N	Y
William Hoyt	N	N	N	N	N	N	N	N	N	Y	N	N	Y	Y	N	Y	N	N	Y
Yangxi	N	Y	Y	N	Y	Y	Y	N	N	Y	N	N	Y	Y	Y	Y	Y	Y	Y
**Completeness of Reporting**	73·4%	86·2%	50·0%	21·3%	27·7%	58·5%	19·1%	13·8%	11·7%	81·9%	14·9%	19·1%	100·0%	100·0%	85·1%	100·0%	62·8%	17·0%	78·7%

Abbreviations - AMD: Age-related macular degeneration, APTOS: Asia Pacific Tele Ophthalmology Society, AV: Arteriovenous, CASIA: Institute of Automation, Chinese Academy of Sciences, CHASE: Child Heart Health Study in England, CLOUD: Contact Lens Anterior Segment- Optical Coherence Tomography Understanding Dataset, DERIVA: Digital Extraction from Retinal Images of Veins and Arteries, DiaRetDB0: Standard Diabetic Retinopathy Database Calibration Level 0, DiaRetDB1: Standard Diabetic Retinopathy Database Calibration Level 1, DR: Diabetic Retinopathy, DR HAGIS: Diabetic Retinopathy, Hypertension, Age-related Macular Degeneration and Glaucoma Images, DRIMDB: Diabetic Retinopathy Image Database, DRIONS: Digital Retinal Images for Optic Nerve Segmentation, DRIVE: Digital Retinal Images for Vessel Extraction, EyePACS: Eye Picture Archive Communication System, FIRE: Fundus Image Registration Dataset, HEI: Hamilton Eye Institute, HRF: High- Resolution Fundus, IDRiD: Indian Diabetic Retinopathy Image Dataset, INSPIRE: Iowa Normative Set for Processing Images of the Retina, JSIEC: Joint Shantou International Eye Centre, LAG: Large-scale Attention based Glaucoma, MACULA: MACulopathy Unveiled by Laminar Analysis, N: No, OCT: Optical Coherence Tomography, OCTRIMA 3D: Optical Coherence Tomography Retinal Image Analysis 3D, ODIR: Ocular Disease Intelligent Recognition, ONHSD: Optic Nerve Head Segmentation Dataset, ORIGA: Online Retinal Fundus Image Dataset for Glaucoma Analysis and Research, PM: Pathological Myopia, RC-RGB-MA: RetinaCheck-

Microaneurysm, RC-SLO: RetinaCheck-Scanning Laser Ophthalmoscopy, RC-SLO-MA: RetinaCheck-Scanning Laser Ophthalmoscopy-Microaneurysm, REFUGE: Retinal Fundus Glaucoma Challenge, REVIEW: Retinal Vessel Image set for Estimation of Widths, RIGA: Retinal fundus Images for Glaucoma Analysis, RITE: Retinal Images vessel Tree Extraction, ROC: Retina Online Challenge, ROCC: Retinal Optical Coherence Tomography Classification Challenge, ROD-REP: Rotterdam Ophthalmic Data Repository, STARE: Structured Analysis of the Retina, VICAVR: VARPA Images for the Computation of the Arterio/Venular Ratio, VOPTICAL: VARPA Optical Dataset, Y: Yes
